# Tongue Strength and Endurance in Healthy Brazilian Adults Measured with the IOWA Oral Performance Instrument

**DOI:** 10.1055/s-0045-1811594

**Published:** 2026-03-11

**Authors:** André Valadares Siqueira, Leandro Castro Velasco, Marina Nahas Dafico Bernardes, Claudiney Candido Costa, Hugo Valter Lisboa Ramos, Noemi Grigoletto De Biase

**Affiliations:** 1 Department of Otorhinolaringology, Centro Estadual de Reabilitação e Readaptação Dr. Henrique Santillo (CRER), Goiânia, GO, Brazil; 2 Universidade Federal de São Paulo (UNIFESP), São Paulo, SP, Brazil; 3 Pontifícia Universidade Católica de São Paulo (PUCSP), São Paulo, SP, Brazil

**Keywords:** tongue strength, Iowa Oral Performance Instrument, deglutition, deglutition disorders

## Abstract

**Introduction:**

The tongue is one of the most important organs in the swallowing process, since impaired tongue function can decrease swallowing effectiveness and safety. Therefore, assessing tongue function is essential to help determine and treat swallowing disorders.

**Objectives:**

To assess tongue strength and endurance in healthy Brazilian adults and to analyze likely variations related to age range and sex.

**Methods:**

In total, 219 individuals > 18 years old, equally divided into age groups (18 to 39 years old, 40 to 60 years old, and > 60 years old, each group comprising 73 individuals), were assessed using the Iowa Oral Performance Instrument (IOPI) to measure tongue strength and endurance.

**Results:**

Tongue strength was significantly lower in older individuals (45.84 kilopascals [kPa]) than in the younger groups (54.16 kPa for young individuals and 54.36 kPa for middle-aged individuals). Similarly, tongue endurance was significantly lower in older individuals. Regarding sex, the assessed parameter only presented significant difference between men and women in the young adult group (men recorded tongue strength 10 kPa higher than that of women).

**Conclusion:**

Normal values for tongue strength in the healthy Brazilian population were established. These values changed based on age group as older individuals presented lower tongue strength and endurance than younger ones. These results highlight the importance of taking age and sex into consideration when assessing tongue strength.

## Introduction

The tongue is an important organ belonging to the stomatognathic system. It plays an important role in chewing, swallowing, sucking, and speech articulation processes along with other structures of this system. 1

The complex organization of its muscle fibers provides the tongue with the ability to move fast, in an effective and precise manner, to significantly contribute to the swallowing process. 2

The tongue plays a crucial role at the initial swallowing process stage, in addition to enabling taste sensation. The pressure exerted by the tongue against the palate contributes to food bolus formation and manipulation, as well as to its safe transport from the oral cavity to the pharynx. 3 4 5 6 7 Tongue weakness may be associated with both impaired bolus formation and increased food debris during meals. Tongue endurance, in turn, is operationally defined as the time interval a given individual can maintain submaximal strength—it generates a fatigue measurement based on certain values. 7

Physiological aging alone can predispose individuals to present swallowing impairment, in addition to several diseases capable of affecting the tongue. 6 Age affects tasks that depend on tongue strength and yet, differences observed in these values between age groups can be attributed to both the size of muscle fibers and sarcopenia rates. 6

Among the most common diseases leading to dysphagia, one finds progressive and/or acute neurogenic disorders, such as stroke, as well as neurodegenerative and muscular diseases. The resulting decrease in tongue strength and endurance compromises bolus formation and makes it hard to propel it into the pharynx. This process leads to varying dysphagia degrees, contributing to dehydration, malnutrition, suffocation and bronchoaspiration pneumonia. 5 8 9 Therefore, it is essential to preserve tongue performance so that it can function properly.

It is essential to assess tongue function to enable both determination and treatment of swallowing disorders. 3 Parameters used to assess tongue performance in clinical practice comprise tongue mobility and strength. 10 11

Tongue strength can be both subjectively and objectively measured. Despite the ease and practicality of the subjective method, the assessment process is based on professionals' experience, besides being somewhat controversial. 12 The objective (quantitative) assessment, in turn, is carried out through instrumental methods to minimize the subjectivity imposed by the background of the examiners. Several instruments have been developed to enable objective tongue strength measurement, such as a mouthpiece equipped with sensors, sensors fixed to the palate, and a bulb filled with fluids. 13

The Iowa Oral Performance Instrument (IOPI) is a portable device capable of measuring tongue strength and endurance. It has been used in recent decades, mainly in the United States, to measure the pressure (in kilopascals [Kpa]) exerted by the tongue, lips and cheeks. 14

Although some studies have documented tongue strength and endurance range in healthy individuals in different parts of the world, there is limited data available in the literature about the Brazilian population. 15 16

## Objectives

To assess tongue strength and endurance to establish normal values for healthy individuals and to analyze the influence of variables, such as sex and age, on tongue strength and resistance in the Brazilian population.

## Methodology

### Study Design

The present study followed observational, descriptive, and cross-sectional research methodology.

### Inclusion Criteria

Population comprising healthy patients and companions, > 18 years old, without any change in the head and neck region or history of surgeries in this region, who agreed to participate in the research and who signed the Free and Informed Consent Form (FICF) ( Supplementary Material Appendix 1 ).

### Exclusion Criteria

The exclusion criteria comprised patients with any type of visual alteration capable of preventing them from seeing the IOPI screen. Individuals with final Eat Assessment Tool (EAT-10) score ≥ 3; patients with oral motor deficiencies (dysphagia, dysarthria, facial or lingual nerve palsy); neurological diseases; trauma; those subjected to radiotherapy and chemotherapy; those who presented any other head and neck disease; and those who underwent tongue, lip and cheek surgery were also excluded from the study.

### Study Sample

Sample size was calculated by taking into consideration IOPI reference values from previous studies conducted with a healthy population. Because these studies presented different results, the weighted value calculated for them, as well as for individual studies, was taken into consideration because this value is not known for the Brazilian population. Thus, the sample size totaled 219 individuals (73 individuals in each group), at 95% confidence level.

The population participating in the present study was stratified based on age groups, and individuals were distributed into the following groups:

- Group 1: young adult individuals in the age group between 18 and 39 years old;
- Group 2: middle-aged individuals in the age group between 40 and 60 years old;
- Group 3: elderly individuals > 60 years old.

### Data Collection

Data collection took place in the outpatient clinic of a Rehabilitation Reference Hospital, from January 2022 to May 2023. It was carried out in a consecutive manner and divided into two different stages:

1. Eating Assessment Tool questionnaire application.
2. Tongue strength and endurance measurement based on the usage of IOPI.

The EAT-10 questionnaire is a subjective assessment tool aimed at identifying the severity of dysphagia symptoms. 17 18

A final EAT-10 score ≥ 3 is considered abnormal (risk of dysphagia); therefore, volunteers who presented abnormal score were excluded from the study. 17

- *Iowa Oral Performance Instrument (IOPI):*

The IOPI is a compact, portable device designed to measure both tongue muscles' strength and endurance based on tongue pressure application against an air bulb connected to a pressure transducer, which, in turn, displays values in kPa.

The light-emitting diode (LED) display provides visual feedback to point out whether the desired pressure level is being applied, while a timer indicates its duration in seconds. 19

- *Tongue strength assessment:*

All tongue measurements were made with participants sitting upright. The IOPI was used to make muscular performance measurements in the anterior part of the tongue.

The bulb was placed in the anterior part of the hard palate and in the posterior part of the central incisor teeth. 19

The examiner held the IOPI tube in the anterior part of the hard palate of the maxilla, as previously specified, and asked the participants to press the bulb with their tongue with maximum force. This was done to measure the maximum strength and resistance values of the tongue. After the measured maximum tongue-pressure value (kPa) was obtained, the participants were allowed to rest for 1 minute and then, the measurement was repeated twice. The highest value (Pmax) among all three measurements was used as study data. 19

- *Tongue endurance assessment:*

Tongue endurance was measured (in seconds) for as long as the IOPI light remained on, at 50% maximum tongue force.

### Data Analysis

The sample size necessary to compare all three groups of individuals (young adults, middle-aged adults and elderly individuals) required 73 individuals in each group. The comparison took into consideration an effect size of 0.2692 (based on the weighted reference value), 5% type I error and 95% test power.

Qualitative variable was expressed as absolute and relative frequency. Quantitative variables were expressed as central tendency and dispersion measures.

The association between qualitative variables was assessed through the Pearson chi-squared test and the Fisher exact test. On the other hand, comparisons among three independent groups were assessed through the Kruskal-Wallis's test, whereas comparisons between two independent groups were assessed through the Mann-Whitney test.

The agreement between tongue strength measurements was determined through intraclass correlation coefficients (ICCs) and their respective 95% confidence interval (95% CIs).

The adopted significance level was 5%. Analyses were carried out in IBM SPSS Statistics for Windows software (IBM Corp.), version 25.0.

## Results

In total, 219 individuals were included in the present study. They were equally divided into 3 groups comprising 73 individuals each, which represented the following age groups: 18 to 39 years old, 40 to 60 years old, and > 60 years old. The variables “sex” and “EAT-10 scores” did not present statistically significant differences among groups ( Table 1 ).

**Table 1:** Study participants' sex, age range, and EAT-10 score

Age group	18–39 years old	4–60 years old	> 60 years old	*p-value*
**Sex**				0.844 ^a^
Female, *n* (%)	44 (60.3)	41 (56.2)	44 (60.3)	
Male, *n* (%)	29 (39.7)	32 (43.8)	29 (39.7)	
**EAT-10**				0.109 ^b^
0	64 (87.7)	64 (87.7)	60 (82.2)	
1	3 (4.1)	8 (11.0)	6 (8.2)	
2	6 (8.2)	1 (1.4)	7 (9.6)	

**Notes** : ^a^ Pearson Chi-squared test; ^b^ Fisher exact test.

### Tongue Strength

Maximum pressure ranged from 17 to 85 kPa. Its approximate mean value reached 51.45 kPa. Group 1, which comprised the youngest participants, recorded a mean maximum pressure of 54.16 kPa (95% CI: 50.89–57.44). Group 2 recorded a mean maximum pressure of 54.36 kPa (95% CI: 51.95–56.76). Finally, group 3, which comprised the oldest participants, recorded a mean maximum pressure of ∼ 45.84 kPa (95% CI: 42.82–48.86), as shown in Table 2 .

**Table 2:** Tongue strength values based on age groups

Age group	All	18–39 years old	40–60 years old	> 60 years old	*p-value* ^a^
	*n =* 219	*n =* 73	*n =* 73	*n =* 73	
**Maximum strength**					< 0.001
Mean ± SD	51.45 ± 13.09	54.16 ± 14.03	54.36 ± 10.31	45.84 ± 12.94	
Minimum– maximum	17–85	18–85	27–78	17–78	
95% CI mean value	49.71–53.19	50.89–57.44	51.95–56.76	42.82–48.86	
**Strength 1**					< 0.001
Mean ± SD	45.90 ± 14.20	49.33 ± 14.11	48.62 ± 12.04	39.74 ± .45	
Minimum– maximum	10–80	17–80	10–73	10–72	
95% CI mean value	(44.00–47.77)	(46.04–52.62)	(45.81–51.42)	(36.37–43.11)	
**Strength 2**					< 0.001
Mean ± SD	48.29 ± 13.92	51.62 ± 13.97	51.07 ± 11.56	42.18 ± 14.16	
Minimum– maximum	12–81	18–81	12–78	12–74	
95% CI mean value	(46.43–50.14)	(48.36–54.88)	(48.37–53.77)	(38.87–45.48)	
**Strength 3**					< 0.001
Mean ± SD)	49.49 ± 13.42	52.15 ± 14.50	52.21 ± 10.80	44.11 ± 13.20	
Minimum– maximum	13–85	13–85	21–74	17–78	
95% CI mean value	(47.70–51.28)	(48.77–55.53)	(49.69–54.73)	(41.03–47.19)	

**Abbreviations** : CI, confidence interval; p _75_ , 75 ^th^ percentile; SD, standard deviation.

**Note** : ^a^ Kruskal-Wallis Test.

There was a statistically significant difference ( *p* < 0.001) between groups 1 and 3, as well as between groups 2 and 3, based on the comparison applied to mean tongue strength values among all 3 groups. However, no significant differences ( *p* = 0.903) were observed between groups 1 and 2 ( Table 2 ).

Three independent tongue strength measures were carried out. The results were expressed as mean value. Agreement among all three measurements was assessed through ICC, which pointed out strong agreement among them (ICC = 0.883; 95%CI 0.856–0.906).

Men in group 1 presented tongue strength significantly higher than that of women ( *p* = 0.001). More specifically, men recorded tongue strength ∼ 10 kPa higher than that observed for women, on average. However, no statistically significant differences in tongue strength were observed between men and women in groups 2 and 3 (> 60 years old), as shown in Table 3 .

**Table 3:** Comparison of maximum tongue strength between women and men based on age group

Tongue strength	Women	Men	*p-value* ^a^
	*n =* 129	*n =* 90	
Group 1	*n* = 44	*n* = 29	0.001
Mean ± SD	49.89 ± 12.85	60.66 ± 13.41	
Group 2	*n* = 41	*n* = 32	0.280
Mean ± SD	53.15 ± 9.73	55.91 ± 10.96	
Group 3	*n* = 44	*n* = 29	0.404
Mean ± SD	44.45 ± 11.90	47.93 ± 14.34	

**Abbreviations** : CI, confidence interval: 75 ^th^ percentile; SD, standard deviation.

**Note** : ^a^ Mann-Whitney test.

Sex-stratified analysis was applied to tongue strength values by comparing different age groups. Statistically significant differences in tongue strength were found in women between groups 1 and 3, as well as between groups 2 and 3. However, no significant differences were found ( *p* = 0.178) between groups 1 and 2 ( Table 4 ). Individuals in the age group > 60 years old recorded lower tongue strength values than those in other age groups.

**Table 4:** Paired comparisons of maximum strength between age groups

	Mean ± SD	*p-value* ^a^
**Women**		
Groups 1 and 2		0.178
Group 1	49.89 ± 12.85	
Group 2	53.15 ± 9.73	
Groups 1 and 3		0.050
Group 1	49.89 ± 12.85	
Group 3	44.45 ± 11.90	
Groups 2 and 3		0.001
Group 2	53.15 ± 9.73	
Group 3	44.45 ± 11.90	
**Men**		
Groups 1 and 2		0.107
Group 1	60.66 ± 13.41	
Group 2	55.91 ± 10.96	
Groups 1 and 3		0.001
Group 1	60.66 ± 13.41	
Group 3	47.93 ± 14.34	
Groups 2 and 3		0.012
Group 2	55.91 ± 10.96	
Group 3	47.93 ± 14.34	

**Abbreviation** : SD, standard deviation.

**Note** : ^a^ Mann-Whitney test.

Tongue strength values recorded for male participants are shown in Table 4 . Based on sample evidence, there were statistically significant differences in tongue strength between groups 1 and 3, as well as between groups 2 and 3. However, no significant difference ( *p* = 0.107) was observed between groups 1 and 2.

### Tongue Endurance

Variation in tongue endurance was observed within the time range from 8 to 180 seconds, with an approximate mean time of 38 seconds. Group 1 recorded a mean endurance of 40.38 seconds, whereas group 2 recorded a mean endurance of 40.74 seconds. Group 3, in turn, recorded a mean endurance of ∼ 35.23s ( Table 5 ).

**Table 5:** Tongue resistance values based on age groups

Age group	All	18–39 years old	40–60 years old	> 60 years old	*p-value* ^a^
	n = 219	n = 73	n = 73	n = 73	
Resistance					< 0.001
Mean ± SD	38.79 ± 22.21	40.38 ± 19.11	40.74 ± 20.21	35.23 ± 26.47	
Minimum– maximum	8–180	12–100	8–100	8–180	
95% CI mean	(35.83–41.74)	(35.92–44.84)	(36.02–45.46)	(29.6–41.41)	

**Abbreviations** : CI, confidence interval; SD, standard deviation.

**Note** : ^a^ Kruskal-Wallis test.

There was a significant difference in tongue endurance between groups 1 and 3 ( *p* = 0.013), as well as between groups 2 and 3 ( *p* = 0.018), based on the comparison among all 3 groups. However, no significant differences ( *p* = 0.983) in this parameter were found between groups 1 and 2 ( Table 6 ).

**Table 6:** Paired comparisons between age groups and tongue resistance

	Mean ± SD)	*p-value* ^a^
Groups 1 and 2		0.983
Group 1	40.38 ± 19.11	
Group 2	40.74 ± 20.21	
Groups 1 and 3		0.013
Group 1	40.38 ± 19.11	
Group 3	35.23 ± 26.47	
Groups 2 and 3		0.018
Group 2	40.74 ± 20.21	
Group 3	35.23 ± 26.47	

**Abbreviation** : SD, standard deviation.

**Note** : ^a^ Mann-Whitney test.

The sex-stratified analysis was applied to tongue endurance values by comparing the different age groups. There were statistically significant differences in tongue endurance ( *p* = 0.015) among women in all 3 age groups ( Table 7 ).

**Table 7:** Comparison of tongue resistance in women among age groups

Age group	18–39 years old	40–60 years old	> 60 years old	*p-value* ^a^
Resistance				0.015
Mean ± SD	41.11 ± 20.34	36.27 ± 21.14	30.36 ± 19.05	

**Abbreviation** : SD, standard deviation.

**Note** : ^a^ Kruskal-Wallis test.

Tongue endurance values observed for men are shown in Table 8 . No statistically significant differences were found between tongue endurance values and age groups ( *p* = 0.165).

**Table 8:** Comparison of tongue resistance in men among age groups

Age group	18–39 years old	40–60 years old	> 60 years old	*p-value* ^a^
Resistance				0.165
Mean ± SD	39.28 ± 17.37	46.47 ± 17.66	42.62 ± 33.93	

**Abbreviation** : SD, standard deviation.

**Note** : ^a^ Kruskal-Wallis test.

## Discussion

Values for both the strength and endurance of the anterior part of the tongues of Brazilian healthy adults were determined in the present study. There was considerable variation in maximum tongue strength values in the analyzed population. Notably, tongue strength values were significantly lower in elderly individuals (45.84 kPa) than in the younger groups (54.16 kPa for young individuals and 54.36 kPa for middle-aged individuals). These results corroborate the assumption that swallowing-associated muscles weaken as individuals grow old, which results in significantly lower muscle strength, mainly in individuals > 60 years old. This reduction in muscle strength can be attributed to muscle atrophy due to muscle mass reduction, as well as to the reduced ability of the human body to both recruit and coordinate motor units due to the aging process, which may have direct effects on muscle weakness. 20 21

Other studies have also supported these findings (i.e., reduced maximum tongue strength in elderly individuals) and highlighted that this pattern seems to result from aging-related effects. 8 10 22 23 24 25 26

Clark et al, conducted a study with the American population and recorded a mean tongue strength of 55.8 kPa for young adults, 62.8 kPa for middle-aged adults and 51.0 kPa for elderly individuals. Although the mean tongue strength observed in the present study has shown slightly different values, both studies have confirmed that mean values recorded for middle-aged adults were similar to those observed for young adults. In addition, elderly individuals tended to present reduced maximum tongue strength. 10 26 A meta-analysis published in 2013 showed that adult individuals < 60 years old presented significantly higher tongue strength than individuals > 60 years old. 15

However, a previous study conducted with typical Portuguese speakers did not identify significant age-related differences. However, it is important emphasizing that this study comprised several participants ( *n* = 75) significantly smaller than that of the present study ( *n* = 219). In addition, the age group > 60 years old was less represented, since it only had 10 participants. Based on these aspects, it is likely that differences between age groups become more evident if one includes a larger number of participants in the oldest age group 16

Regarding sex, only the group of young adults recorded significant difference in tongue strength between men and women in the present study – the mean pressure was ∼ 10 kPa higher in men. This outcome is in line with Utanohara et al., who also found similar results and evidenced higher tongue strength in men than in adult women. 5 However, such a difference was not observed in elderly individuals. Moreover, other studies have also corroborated this disparity in tongue strength between men and women. 27 28

A plausible explanation for this sex-related disparity may lie on anatomical differences between both sexes. Men often present higher muscle mass and it could result in increased strength. However, it is important to emphasize that not all studies observed the same results. Vitorino, Vanderwegen et al., and Clark et al., did not find significant differences in tongue strength between sexes.

Evidence available on the impact of age and sex on tongue endurance remains limited and, for the most part, it did not present any significant association between these factors. Based on the systematic review conducted by Adams et al., there was no statistically significant difference in tongue endurance between men and women in all age groups. In addition, age effect on tongue endurance was not observed, as shown in the aforementioned review. These results were in line with other studies that also observed lack of influence of sex and age on tongue endurance. 16 22 23 29

However, findings in the present study differed from some previously reported ones. It was observed that, just as tongue strength, tongue endurance was significantly lower in elderly individuals than in younger ones (< 60 years old). According to Deschenes, this finding is justified by the fact that sarcopenia is not only characterized by loss of type II fibers (rapid contraction), since there are other associated physiological mechanisms, such as denervation followed by loss of motor units and, consequently, of muscle fibers, but also by metabolic changes with subsequent decrease in the production of anabolic hormones, increased catabolic agents, and impaired muscle strength and endurance as individuals age. 30

Kays et al. observed results similar to the ones recorded in the present study, such as declined endurance in the anterior part of the tongue as individuals age, which can be justified by the fact that the anterior lingual region is mostly formed by fibers susceptible to type II fatigue. These fibers provide less resistance to the anterior region of the tongue as individuals age. 31

The present study has evidenced that the IOPI is a good tool to achieve objective tongue-strength assessment. Three independent tongue strength measurements were carried out, and they presented excellent agreement based on the ICC. Previous studies have evidenced high reliability in isometric tongue-strength measurements based on IOPI, as well as high-to-very-high correlation coefficients. 15 22 23

The present study is a pioneering contribution, as well as a comprehensive research work, conducted in Brazil, to determine normal tongue strength values in the Brazilian adult population. Unlike the previous study, which comprised a small sample, the present study adopted a comprehensive and careful approach, as well as a proper sample calculation method and significant sample size. Therefore, its results provided a reliable and representative tongue strength scenario for the Brazilian healthy adult population.

However, it is important to acknowledge and emphasize the limitations of the present study. One of the most important limitations lies on the geographical restriction for data collection, since the present research was exclusively conducted in the city of Goiânia, state of Goiás, Brazil. Besides demographic features, other factors, such as socioeconomic, cultural, ethnic and lifestyle conditions, as well as individuals' use of medication and medical conditions, were not taken into consideration.

Accordingly, it is necessary to interpret the current findings with caution, as well as to take into consideration the need of conducting future research focused on addressing a more diverse and representative fraction of the Brazilian population.

## Conclusion

The present study identified the mean tongue strength and endurance values in healthy adult individuals, based on participants' age and sex. Moreover, men recorded tongue strength and endurance values higher than those observed for women among young individuals, whereas these values decreased as individuals aged.
