# Supplementary material for: Tongue Strength and Endurance in Healthy Brazilian Adults Measured with the IOWA Oral Performance Instrument
Source: Int Arch Otorhinolaryngol. 2026 Mar 11;30(1):1–8. doi: 10.1055/s-0045-1811594 (PMC12978957; doi:10.1055/s-0045-1811594)
Supplement: Supplementary file 1 — Supplementary Material [file 10-1055-s-0045-1811594-s252005.pdf]

CONSENT FORM - STATE CENTER FOR REHABILITATION AND READAPTATION DR. HENRIQUE SANTILLO – GOIANA ASSOCIATION FOR INTEGRATION AND REHABILITATION.

You are being invited to participate in the subproject: “Tongue Strength and Endurance in Healthy Brazilian Adults Measured with the IOWA Oral Performance Instrument (IOPI)”, of the research: “STUDY OF DISORDERS AND REHABILITATION OF THE UPPER AERODIGESTIVE TRACT.” The objective of the study is to evaluate tongue strength and resistance in healthy adults and to analyze possible variations in relation to age group, sex, race, body mass and lifestyle habits, contributing to the provision of data for the development of future studies in the area. The research will be carried out through the application of the Iowa Oral Performance Instrument (IOPI), which is a device used to evaluate tongue strength and resistance, and a self-administered questionnaire to healthy adults. It is a non-invasive research, as there will be no collection of biological material. There will be no cost to the study participant and if you refuse or withdraw from participating there will be no penalty of any kind. However, your participation is very important for the execution of the study and the confidentiality and privacy of the information you provide will be guaranteed. Any data that could identify you will be omitted in the disclosure of the research results and the material will be stored in a safe place. The results will be published in scientific journals in the health area. If you have any questions, you can ask me.

CONSENT TO THE PATIENT PARTICIPATION

I, ____, agree to participate in the subproject: ““STUDY OF DISORDERS AND REHABILITATION OF THE UPPER AERODIGESTIVE TRACT.” I declare that I have been duly informed and clarified by the researcher ____ about the objectives of the research, the procedures involved in it, as well as the possible risks and benefits involved in my participation. I understand that I can say “yes” and participate, but that, at any time, I can say “no” and withdraw without penalty. I was given the opportunity to ask questions and was given collect telephone numbers to contact, in case I have any questions.

Place and date

____.

Fingerprint Signature:

♦ Name and Signature of the researcher in charge:

____

We witnessed the request for consent, clarifications about the research and the subject's acceptance to participate.

Witnesses (not linked to the research team):

Name: ____________________________________

Signature: _________________________________

Name: ____________________________________

Signature:_________________________________

FREE AND INFORMED CONSENT FORM STATE CENTER FOR REHABILITATION AND READAPTATION DR. HENRIQUE SANTILLO – GOIANA REHABILITATION ASSOCIATION.

You, ____, are being invited to authorize, as a volunteer, your participation in a research study. My name is ____, I am the principal investigator and my field of expertise is medicine. After carefully reading this document and being informed about the following information, if you agree to take part in the study, please sign at the end of this document, which is in two copies. One is yours and the other is the principal investigator's. If you have any questions about the research, you may contact the principal investigator, Leandro Castro Velasco, at (062) 3232-3232. If you have any questions about your rights as a participant in this research study, you may contact the Leide das Neves Ferreira Center for Excellence in Teaching, Research and Projects CEEEPP-LNF/SES/GO: Rua 26, n. 521, Jardim Santo Antônio, Goiânia-GO, CEP: 74853-070. Phone: 62)3201-3408. Email: cep.ceepp@gmail.com.

INCLUSION CRITERIA FOR PARTICIPATION IN THE RESEARCH:
• Healthy adult patient.
• Age over 18 years.
• Have agreed and signed the Consent Form.

EXCLUSION CRITERIA FOR PARTICIPATION IN THE RESEARCH:
• Individuals with oral motor deficiencies (dysphagia, dysarthria, facial or lingual nerve paralysis), neurological diseases, trauma, radiotherapy and chemotherapy or any other head and neck disease and those who have undergone surgery on the tongue, lip and cheek.
• Forms that have erasures or items marked incorrectly;
• Forms that are not fully answered;
• If the guardian or the patient refuses to participate in the project.

INFORMATION ABOUT THE RESEARCH:

Research Title: "STUDY OF DISORDERS AND REHABILITATION OF THE UPPER AERODIGESTIVE TRACT." Responsible researchers: Claudiney Candido Costa, Hugo Valter Lisboa Ramos and Mikhael Romanholo El Cheikh.

Subproject Title: "Tongue Strength and Endurance in Healthy Brazilian Adults Measured with the IOWA Oral Performance Instrument (IOPI)".
Responsible researcher: Leandro Castro Velasco.
Contact telephone numbers: (62) 3232-3232.
E-mail: lcvelasco@gmail.com.
Responsibility: otorhinolaryngologist, coordinator of the subproject, final writing of the text,

publication.

Principal Investigator: André Valadares Siqueira.

Contact telephone numbers: (62) 3232-3232.

E-mail: andrevaladares@hotmail.com.

Responsibility: otorhinolaryngologist, master's student, final draft of the text, publication.

Researcher: Marina Nahas Dafico Bernardes

Contact telephone number: (62) 98143-5227

E-mail: med.marinanahas@gmail.com

Responsibility: resident physician in Otorhinolaryngology at CRER, authorized to apply the consent forms, perform the exams, perform statistical analysis, final draft of the text, publication.

Proposing institution: CRER. Location where the evaluation of speech, breathing and swallowing disorders will be performed, including the IOPI test, as well as the storage of data contained in the medical records and outpatient monitoring at the service.

25 CEEEPP-LNF/SES/GO: Street 26, no. 521, Santo Antônio Garden, Goiânia-GO, Zip Code: 74853-070. Phone: (62)3201-3408. Email: cep.ceepp@gmail.com.

The research aims to evaluate the strength and resistance of the tongue in healthy adults and analyze possible variations in relation to age group, sex, race, body mass index and lifestyle habits. To this end, some questionnaires with objective questions will be applied, which must be answered honestly, and the strength and resistance of the tongue will be measured with the IOPI. To measure the maximum strength and resistance of the tongue, the examiner will hold the IOPI tube and place the bulb on the tongue in the area of the anterior lingual hard palate of the maxilla and ask the subject to press the bulb using the tongue with maximum force for two seconds. After obtaining the measured value of maximum tongue strength (Pka), the subject will rest for one minute and then repeat the measurement two additional times. The highest value measured will be used as data. Tongue resistance will be measured in seconds for the period of time in which the IOPI light is maintained at 50% of maximum tongue strength.

Participation in the research will not cost you anything, nor will there be any type of payment or financial reward for your participation. We guarantee clarifications at any time. There is no financial value, to be received or paid, for your participation. By participating in the research, the patient will be contributing to the performance of a very important study, and may help to propose measures to perform the diagnosis more quickly and the most appropriate treatment of the alterations that are being studied. You will have access to all the results of the exams at any time, as well as access to the final result of the research, if this is of interest to you. The patient is free to refuse to participate in the study, as well as to withdraw consent, without any prejudice to the continuity of the usual monitoring/treatment. The volunteer is guaranteed complete confidentiality, meaning that he/she will not be identified when answering the questionnaires. The data collected will be used only for this research and will not be stored for future studies. The results of the exams, as well as any evaluation of the patient, will only be the responsibility of the researchers involved in the project and the professionals who may have a relationship of care and/or assistance with the patient. Access to third parties (followers, employers, hierarchical superiors) will not be permitted, ensuring protection against any type of discrimination and/or stigmatization. There is a risk of loss of anonymity of the participants (if any form is misplaced or lost, since the people who get the form may discover the participant's health problems), with

possible damage to the public image of the participant and risk of moral damages. To avoid this type of problem, the researchers will be careful and keep the forms in a locked cabinet, which only they have access to.

Name and Signature of the researcher ____

CONSENT TO PARTICIPATE AS A SUBJECT

I, ____, ID/CPF/medical record number ____, undersigned, agree to participate in the study "EVALUATION OF TONGUE STRENGTH AND RESISTANCE, USING THE IOWA ORAL PERFORMANCE INSTRUMENT (IOPI), IN HEALTHY ADULT PATIENTS.", under the responsibility of Dr. Leandro Castro Velasco, as a voluntary subject. I have been duly informed and clarified by the researcher ____ about the research, the procedures involved in it, as well as the possible risks and benefits arising from my participation. I have been assured that I may withdraw my consent at any time, without this leading to any penalty or interruption of my monitoring/care/treatment.

Place and date

____.

Name and signature of the subject:

____

Fingerprint signature:

Name and signature of the researcher in charge:

We witnessed the request for consent, clarifications about the research and the subject's acceptance to participate.

Witnesses (not linked to the research team):

Name: ______________________________________

Signature: ___________________________________

Name: ______________________________________

Signature: ___________________________________
